# Update of Aging Hallmarks in Idiopathic Pulmonary Fibrosis

**DOI:** 10.3390/cells14030222

**Published:** 2025-02-05

**Authors:** Ana Lilia Torres-Machorro, Ángeles García-Vicente, Marco Espina-Ordoñez, Erika Luis-García, Miguel Negreros, Iliana Herrera, Carina Becerril, Fernanda Toscano, Jose Cisneros, Mariel Maldonado

**Affiliations:** 1 Laboratorio de Biología Celular, Instituto Nacional de Enfermedades Respiratorias Ismael Cosío Villegas, Ciudad de México 14080, Mexico; ana.torres@iner.gob.mx (A.L.T.-M.);; 2 Facultad de Ciencias, Universidad Nacional Autónoma de México, Ciudad de México 04510, Mexico; angelesgvic@gmail.com; 3 Posgrado en Ciencias Biomédicas, Unidad de Posgrado, Ciudad Universitaria, Ciudad de México 04510, Mexico; 4 Departamento de Investigación en Fibrosis Pulmonar, Instituto Nacional de Enfermedades Respiratorias Ismael Cosío Villegas, Ciudad de México 14080, Mexico; canoscream@ciencias.unam.mx (M.E.-O.); jcisneros828@gmail.com (J.C.); 5 Posgrado en Ciencias Biológicas, Unidad de Posgrado, Ciudad Universitaria, Ciudad de México 04510, Mexico; 6 Clínica de Vasculitis Sistémicas Primarias, Instituto Nacional de Enfermedades Respiratorias Ismael Cosío Villegas, Ciudad de México 14080, Mexico; mik_o16@hotmail.com; 7 Laboratorio de Biopatología Pulmonar, Instituto Nacional de Enfermedades Respiratorias Ismael Cosío Villegas, Ciudad de México 14080, Mexico

**Keywords:** idiopathic pulmonary fibrosis, aging hallmarks, mechanical alterations, dysbiosis, inflammaging, alternative splicing

## Abstract

Idiopathic Pulmonary Fibrosis (IPF) is an epithelial-driven interstitial lung disease of unknown etiology characterized by the excessive proliferation of fibroblast populations that synthesize large amounts of extracellular matrix. In this devastating disorder, all aging hallmarks appear prematurely or are altered. This review highlights key findings about IPF characteristics recently recognized as hallmarks of aging, including mechanical alterations, inflammaging, dysbiosis, alternative splicing, and disabled macroautophagy. It also revisits the classic hallmarks of aging, which encompass stem cell exhaustion, cellular senescence, and altered intercellular communication. Enhancing our understanding of the fundamental processes that underlie the altered hallmarks of aging in IPF may facilitate the development of innovative experimental strategies to improve therapeutic outcomes.

## 1. Introduction

Idiopathic Pulmonary Fibrosis (IPF), the most aggressive interstitial lung disease of unknown etiology, is an epithelial-driven disorder where fibroblast populations proliferate and differentiate, completely remodeling lung architecture [1,2]. As a condition predominantly affecting older people, IPF displays all the original and newly added hallmarks of aging in a manner that is either premature or altered [3].

The nine classic hallmarks of aging, delineated in a seminal publication over a decade ago, encompass genomic instability, telomere attrition, epigenetic alterations, loss of proteostasis, deregulated nutrient sensing, mitochondrial dysfunction, cellular senescence, stem cell exhaustion, and altered intercellular communication [4]. Recent advances have distinguished disabled macroautophagy from loss of proteostasis, while chronic inflammation and dysbiosis were added as new hallmarks [5]. Other newly proposed hallmarks of aging include alternative splicing and changes in mechanical properties [6]. This review provides an update on the significant advancements in our understanding of the aging hallmarks within the context of IPF, beginning with insights into the additional or complementary hallmarks and subsequently updating the classical ones alongside some of the latest promising therapeutic options undergoing clinical trials. Most hallmarks are profoundly interconnected, cooperatively affecting single processes.

## 2. Complementary Hallmarks

### 2.1. Mechanical Alterations

Over the past decades, the role of mechanical forces has gradually emerged as a crucial factor influencing cellular behavior, with relevance to soft, elastic tissues such as the lungs. Fibroblasts, beyond their role in synthesizing the extracellular matrix (ECM), are also influenced by the mechanical properties of the ECM, including stiffness, viscoelasticity, topography, tensile stress, and shear modulus, among others. This bidirectional interaction between cells and ECM has gained prominence as a fundamental area of research in fibrotic diseases. IPF research is no exception, with numerous studies examining how healthy versus fibrotic ECMs modify cell functions [7,8,9].

As aging progresses, organs often accumulate damaged ECM, resulting in increased stiffness due to glycation, cross-linking, and the accumulation of their components. This process is called Fibroaging [10]. Evidence suggests that, while the collagen and polysaccharide content may not differ significantly between healthy and fibrotic lungs, the organization of these ECM components does. As a result, the tissue’s mechanical properties change, prompting fibroblasts to modify their behavior [8]. The extracellular products of these senescent fibroblasts, collectively known as the Senescence-Associated Secretory Phenotype (SASP), can increase matrix stiffness and complicate cell communication [11]. Additionally, the organization and composition of the ECM are crucial for the migration and gene expression of epithelial cells [9].

Age-related changes in lung ECM include elevated COL6A2, which may impair pulmonary elasticity. An increased COL14A1 in the bronchial epithelium suggests a role in maintaining epithelial composition. Increased levels of the proteoglycan LUM in aged lungs may represent a compensatory response to increased injury, underscoring its role in wound healing and collagen organization. These findings emphasize key ECM adaptations during lung aging and their potential role in chronic lung diseases [12].

Mechanical features in ECM are closely linked to cellular response, enabling cells to sense and adapt to various mechanical stimuli. In the lung, epithelial cells respond to both stiffness and stretch stimuli. Epithelial cells cultured on a special device to simulate stretch entered a senescent state observed by senescence-associated β-Galactosidase activity (SA-βGal) and cell cycle arrest. In this situation, cells only received the mechanical force of stretch instead of an exogenous DNA stressor. In addition, fibroblasts stimulated with the supernatant of stretched epithelial cells acquired a migratory phenotype expressing alpha-Smooth Muscle Actin (αSMA) [13]. These data relate to the interconnection between the hallmarks of senescence and mechanical alterations.

Integrins are transmembrane heterodimeric proteins that sense stiffness. Their inhibition, specifically of the monomer αv, decreased lung fibrosis [14]. Consequently, new treatments have been designed and tested with cohorts of patients. For example, INTEGRIS-IPF is a clinical trial with PLN-74809 (bexotegrast), an inhibitor of αvβ1 and αvβ6 integrins. In in vitro assays, this inhibitor decreases fibrotic-related molecules such as *COL1A1*, *COL3A1*, *COL1A2*, *ACTA2*, and *SERPINE1*, among others [15]. It is safe and tolerable for patients, diminishing circulating levels of β6 integrin and N-terminal type III collagen propeptide [16]. A slower decline in the forced vital capacity (FVC), compared to in placebo patients, was observed. Interestingly, an increase in the patient’s FVC was observed with the highest dose [16]. Some authors suggest that the profibrotic effects of integrins are due to the activation of the Transforming Growth Factor Beta (TGFβ) [17]; however, the levels of integrins and collagen fragments in blood might have unknown implications. Future basic research will uncover the complete targets of this inhibitor, as well as the other mechanical alterations in IPF.

To analyze the participation of the ECM composition, structure, and mechanical properties in the behavior of lung cells, experimental strategies have been developed to study them in microenvironments with controlled mechanics. Natural or synthetic hydrogels have been recently reviewed [18] and are the most affordable and widespread. Natural hydrogels are elaborated with collagen, matrigel, or gelatin methacrylate (GelMa), which is the product of the reaction of methacrylic anhydride and the hydrolyzed collagen [19,20]. On the other hand, synthetic hydrogels are more stable, and stiffness is strictly controlled. These hydrogels can be prepared with polyethylene glycol, polyacrylamide, or Polydimethylsiloxane (PDMS) and include a coating of different ECM proteins [21,22]. In addition, 3D co-culture of fibroblast and epithelial cells in collagen matrices [23] and cell culture on Precision-Cut Lung Slices (PCLS) have been proposed. The use of these novel cell culture strategies is undergoing exponential growth. This is demonstrated by Pubmed search results, which consist of 2142 papers, containing 291 dedicated to IPF.

Some researchers state that the ECM is more important in the progression of the fibrotic condition than the fibroblast origin, where they believe that the translational regulation by miRNAs is more critical than the transcriptional regulation [7]. There is still much to study regarding the effects of mechanical stimuli from the ECM on the pathology of IPF and individually on the behavior of each cell type. However, developing these three-dimensional culture and co-culture systems that better represent cell–cell and cell–ECM interactions in the in vivo environment was an essential step toward understanding both the pathology and the role of the ECM.

### 2.2. Alternative Splicing

Dysregulated Alternative Splicing (AS) is an aging hallmark that can also arise from other hallmarks, including epigenetic dysregulation, genomic instability, and loss of proteostasis. The distorted DNA methylation in IPF [24] is an example of epigenetic dysregulation. The genetic mutations altering coding regions [5] and dysregulation of splicing factors like the SR protein SF2/ASF [25] and the Epithelial Splicing Regulatory Protein 1 (ESRP1) [26] are examples of genomic instability and loss of proteostasis, respectively.

Two genome-wide RNA-seq reports identified multiple transcripts that were alternatively spliced in IPF [27,28]. Deng and collaborators found 122 non-differentially expressed genes with high splicing isoform exchange when comparing control and IPF samples [27]. Only three alternatively spliced genes in IPF were confirmed in this work [27] (Table 1). Nance and collaborators identified 436 unique genes with significant differential splicing events in at least one exonic region [28]. They enlisted the top genes with differentially spliced exons that included *RPS24*, *DLC1*, *COL3A1*, *ZFP36L1*, *GM2A*, and *TRA2B*, as well as the PCR-validated genes *COL6A3* and *POSTN* [28]. Table 1 lists the confirmed IPF alternatively spliced genes, their expression levels, and their presumed protein function in IPF. It also includes data from individual reports of IPF spliced genes like *esAGER* [29] and *FN1-EDA* [30].

Candidate genes that could have altered splicing in IPF were also identified throughout this compilation effort. These genes were identified in reports of the alternative splicing in pulmonary fibrosis of known etiologies *CD44* [31], *RXFP1* [32], and *CD146* [33], a pulmonary fibrosis mouse model (*FGFR2*) [26], and a genetic link between COVID-19 and IPF (*DP99* and *ATP11A*) [34,35]. Research on the AS of these genes could identify other alterations in IPF.

While multiple splicing isoforms have been discovered and studied in other diseases [36,37,38], only a few alternatively spliced genes characteristic of IPF have been described (Table 1). Moreover, most genes lack confirmation of expression of the corresponding protein isoforms. Therefore, understanding this complex disease requires validating genome-wide predicted variants and functional assays for confirmed IPF mRNA variants.

**Table 1:** Confirmed genes with alternative splicing in IPF. α The Ensembl-reported isoform codes for a predicted 3177-amino-acid (aac) protein. β The only Ensembl-reported isoform lacking exon 4 codes for a predicted 638 aa protein. γ Our verification and comparison of the two reported Ensembl reference numbers for the *TOM1L1* splicing isoforms did not find differences at exon 6; instead, the difference is localized at exon 2. The predicted proteins derived from the Ensembl [39] transcripts have either the presence (ENST00000348161) or absence (ENST00000445275) of SH2 and SH3 domains in the *TOM1L1* isoforms. δ Even though both reported Ensembl transcripts differ at the 5′ external non-translated sequences of the mRNA, further differences are found in the coding region, leading to predicted proteins of differing sizes (259 (ENST00000369306) vs. 81 aac (ENST00000428634)). The short predicted protein is probably non-functional because it lacks all the C-terminal regions containing the peroxisome targeting and protein–protein interaction domains [40,41,42,43,44,45,46].

Gene	Isoforms	Ensemble Reference	Function	In IPF vs. Controls	Domain Lost	Domain Gained	Additional Information	Ref.
AGER	Advanced Glycosylation End-Product Receptor	ENST00000375076.9	Immuno-globulin superfamily cell-surface receptor					[40]
	Endogenous secretory (es)	No	Secreted, non-signaling receptor	Down	Trans-membrane		Considered to have an anti-inflammatory function.	[29]
POSTN	Periostin, osteoblast-specific factor	ENST00000379747.9	ECM protein involved in tissue development and regeneration					[41]
	Shorter variant	No	Proposed reduced ECM protein binding and altered cell invasiveness	Down	C-ter portion		Exon 21 is more likely to be spliced out in IPF.	[28]
COL6A3	Collagen Type VI Alpha 3 Chain, α	ENST00000295550.9	Cell adhesion and integrin signaling				Exon 4 inclusion was previously associated with colon and pancreatic cancer.	[42,43]
	Shorter variant	ENST00000347401.8		Down			Exon 4 is more likely to be spliced out in IPF.	[28]
TOM1L1	Target Of Myb1-Like 1 Membrane Trafficking Protein	ENST00000445275	Probable adapter protein, clathrin, and kinase binding activity	Down		Exon 6, γ		[27,44]
	Shorter variant	ENST00000348161		Upregulated	Exon 6		Exon 6 skipping is more likely in IPF.	
CMTM4	CKLF-Like MARVEL Transmembrane Domain Containing 4	ENST00000330687	Chemokine-like factor, cell growth, and cell cycle regulation	Upregulated		C-ter region	Alternative 3′ splice sites generate two protein isoforms with 26aac difference.	[27,45]
	Shorter variant	ENST00000394106	Believed to contribute to stress-induced senescence	Down	C-ter region			[27]
PEX11B	Peroxisomal Biogenesis Factor 11 Beta	ENST00000369306	Peroxisome metabolic pathway and oxidative stress protection	Down		5′ end, δ		[27,46]
	Shorter variant	ENST00000428634	May contribute to peroxisomal dysfunction, generating oxidative stress	Upregulated	5′ end		Truncated 5′ end by alternative promoter.	[27]
SCL38A10	Solute carrier family 38 member 10	ENST00000374759	Predicted involvement in amino acid transmembrane transport	Down		N-ter region		[27,39]
		ENST00000374759_N	Novel transcript	Upregulated	Exon 2	N-ter region		[27]
	Truncated 5′ end	ENST00000288439		Down	N-ter region			[27,39]
		ENST00000288439_N	Novel transcript	Upregulated	N-ter regionAnd exon 2			[27]
FN1	Fibronectin I		Cell adhesion and migration					
	EDA (extra type III domain A)	ENST00000354785.11	Important for fibroblast activation	Upregulated		Type III domain A		[30]

### 2.3. Inflammaging

One complementary hallmark of aging is chronic inflammation or inflammaging, a systemic sterile inflammation associated with fewer naïve T cells, increased memory T cells, and a wide range of age-related diseases [47]. This process is interconnected with other hallmarks, such as senescence, through the secretion of cytokines belonging to the SASP [47]. These interrelations among immune dysregulation, cellular senescence, inflammation, and aging have previously been associated with IPF [48,49].

The role of inflammation in IPF remains controversial, with some authors considering it as an epiphenomenon of fibrosis, occurring independently of fibrotic remodeling [50]. This perspective is supported by the inadequacy of immunosuppressant treatments in IPF patients [1,51]. Nevertheless, evidence points to an aberrant inflammatory response in IPF involving immune cells and the cytokines they secrete, as described below.

#### 2.3.1. Innate Immunity and Fibrocytes

Inflammatory cells, including alveolar macrophages, neutrophils, and lymphocytes, are present in the lungs of IPF patients. Through crosstalk with lung fibroblasts, these cells may variably contribute to the pathogenesis of IPF. The presence of immune cells in IPF lungs was supported by necroptosis, a pro-inflammatory type of programmed cell death associated with immune infiltration [52].

The cytokine CCL2, also known as MCP-1 (Monocyte Chemoattractant Protein-1), regulates monocyte migration and is elevated in the serum of IPF patients. Nevertheless, inhibiting CCL2 has no clinical benefit for patients [53,54]. There are several monocyte subsets classified according to the surface markers. Relative to controls, an increased proportion of the CD64hi monocytes in IPF may promote a significant response to type I interferon (IFN) and perhaps lead to chronic inflammation and fibrosis [55].

Fibrocytes are derived from monocytes and are fibroblast precursors. Fibrocytes are present in the lungs, and increased levels were observed in the peripheral blood of IPF patients [56,57]. Some evidence suggests that fibrocytes contribute to the fibrotic process. For example, it was demonstrated that the differentiation of fibrocytes from monocytes could be promoted by increased cofilin-1, a blood characteristic of IPF [58]. In a recent clinical trial, the use of sirolimus, a mammalian Target of Rapamycin (mTOR) inhibitor, reduced the circulating number of fibrocytes in IPF patients [59]. However, fibrocytes are not yet considered prognosticators of the disease [60,61]. Thus, the potential contribution of fibrocytes to IPF and inflammation needs additional research.

Macrophages are phagocytic cells critical for tissue remodeling and immune/inflammatory response. Several studies have shown that macrophages release significant numbers of cytokines that regulate mechanisms leading to pulmonary fibrosis. CCL17 is a macrophage-derived cytokine that mediates fibroblast activation through interaction with CCR4. Recently, it was demonstrated that CCL17 is increased in IPF lungs and the bleomycin mouse model [62]. This axis is a potential target to attenuate fibrosis progression.

An imbalance between M1 (pro-inflammatory) and M2 (anti-inflammatory and profibrotic) macrophages has been shown in IPF. Recently, it was demonstrated that the SHP-1 agonist downregulated the IPF macrophage polarization, ameliorating pulmonary fibrosis. The observed effect was probably mediated by reduced STAT3/NF-κB signaling and fibroblast–myofibroblast transition [63]. Inhibition of the M2 macrophage polarization also mitigated the bleomycin-induced pulmonary fibrosis via downregulation of the CYTL1 and TGF-β/CCN2 axis in mice [64]. In summary, the blockage of M2 macrophage polarization could serve as a target to alleviate the lung injury present in IPF.

Neutrophils are other cells of the innate immune system that contribute to inflammation. In IPF patients, neutrophils and their chemoattractant IL-8 increase in Bronchoalveolar Lavage (BAL) [65]. A reduction in neutrophil chemotaxis contributed to a decrease in the disease severity when mice were treated with nintedanib in the pulmonary fibrosis mouse model [66]. Neutrophil elastase, a toxic product of neutrophils, contributes to pulmonary fibrosis, whereas its antagonist attenuated fibrosis in several pulmonary fibrosis models [67].

Eosinophils are a scarce type of leukocyte (<5%) derived from bone marrow hematopoietic stem cells and a source of IL-13. Their expression is upregulated in the BAL and lung tissue of IPF patients. Additionally, IL-13 stimulates proliferation and extracellular matrix protein production in lung fibroblasts [68].

#### 2.3.2. Adaptative Immunity

The adaptative immune system also contributes to inflammation in IPF. For example, the CD4+ T lymphocytes secrete multiple cytokines. Th1 cells release IL-2, IL-12, interferon-gamma (IFN-γ), and tumor necrosis factor (TNF). This phenotype may be protective and contribute to inflammation in the lungs. Conversely, Th2 cells secrete IL-4, IL-5, IL-6, IL-10, and IL-13, promoting a fibrotic response [69]. In the context of IPF, Th9 cells have a dual role in inflammation by secreting IL-19, activating lung fibroblasts, and secreting IL-4 and inducing the differentiation of Th0 to Th2 cells [70]. The Th17 CD4+ cells contribute to tissue inflammation by producing IL-17A, IL-21, and IL-22. IL-17A is known to sustain lung inflammation [69], enhance fibroblast proliferation and collagen synthesis, and stimulate the secretion of TGF-β and IL-6 from lung fibroblasts [71].

The CD8^+^ T cell number in the BAL of IPF patients positively correlated with the severity of pulmonary fibrosis [72]. In lung samples derived from IPF patients, the percentage of this T cell subpopulation was increased, and their transcriptional signature and signaling pathways were associated with fibrosis, suggesting a critical role in IPF progression [73]. Therefore, several studies have identified genes regulating T cells with potential therapeutic usages for IPF, including the Novel Kinase 1 (NUAK1) [74], STEAP2 [75], SPP1, IGF1, ASPN, and KLHL13 [76].

The B cells, a humoral component of the adaptative immune system, are increased in the lung and BALs [77] of IPF patients. Recently, a bioinformatic study revealed immune infiltration and upregulated memory B cells in IPF [78]. Another study showed that B cells could induce fibroblast migration and activation [79]. Thus, the accumulation of B cells in IPF patients may promote or sustain ongoing inflammation. Supporting the above, bortezomib-mediated depletion of plasma cells (antibody producers) effectively inhibited pulmonary fibrosis [80]. However, B cell inhibition using anti-CD20 did not reduce bleomycin-induced lung fibrosis [80].

The crosstalk between lung fibroblasts and immune cells has emerged as a critical process in promoting an efficient wound-healing response. T cells, B cells, macrophages, and dendritic cells accumulate within specific foci in the lung tissue adjacent to areas of active tissue fibrosis [78].

Finally, IPF lung fibroblasts release pro-apoptotic molecules, such as pro-caspase 3, cytochrome C, HIF-1α, HTRA2-OMI, and TNFR1, that reduce migration (via RhoA/ROCK) and induce apoptosis in T cells. This mechanism could explain the absence of lymphocytes inside fibroblast foci in IPF [81]. The text above underscores the concept of immune cell crosstalk between lymphocytes and fibroblasts; however, the precise mechanisms governing this interaction remain poorly understood.

In summary, the aberrant activity of the innate and adaptive inflammatory processes causes inflammatory changes that occur independently of the primary fibrotic remodeling process in IPF.

### 2.4. Dysbiosis

Growing evidence suggests that variations in the composition of the intestinal and pulmonary microbiota are directly associated with the progression of chronic respiratory diseases. However, the causal relationship between microbiota alterations and disease progression remains unclear. Dysbiosis, an imbalance in microbiota, has emerged as a salient topic in aging-related pathologies, including IPF. The role of the lung microbiome as a trigger for the development and progression of lung fibrosis has recently been investigated.

In the airways of healthy individuals, the microbiome is primarily composed of four groups: Bacteroidetes, which includes *Prevotella* spp.; Firmicutes, such as *Streptococcus* and *Veillonella*; and, to a lesser extent, Proteobacteria and Actinobacteria. The composition of the lung microbiome is influenced by three main factors: microaspiration of gastric contents, microbial elimination, and the local microbial growth environment. These factors regulate microbes’ influx, efflux, and reproduction rates, which are altered during pathological conditions [82,83].

The hypothesis that microbial activity influences the progression of IPF is supported by evidence showing that antibiotic treatment can reduce mortality rates, increase life expectancy, and decrease the need for oxygen therapy. However, this treatment did not significantly improve the lung function [83]. Several studies have demonstrated alterations and higher overall bacterial loads in IPF BALs, including *Haemophilus*, *Streptococcus*, *Neisseria*, and *Veillonella* spp. loads [84]. Friaza et al. identified bacterial sequences corresponding to *Streptococcus*, *Neisseria*, and *Actinobacterium* spp. in a metagenomic study in BALs from 17 IPF patients. Interestingly, bacterial DNA was not detected in five out of eight patients colonized with *Pneumocystis jirovecii*, suggesting that the presence of fungi may alter the colonization of specific bacterial groups in the airways. These findings suggest a direct association between the abundance of specific bacteria, including *Streptococcus* and *Veillonella*, as well as *Staphylococcus* and *Prevotella*, and the advancement of the disease, along with diminished life expectancy [82,85,86,87,88].

Han et al. observed a link between the lung microbiome and IPF progression. By Correlating Outcomes with biochemical Markers to Estimate Time progression (COMET), they identified a significant relationship between the relative abundance of *Streptococcus* and *Staphylococcus* and disease development [89]. Similarly, Huang et al. uncovered immune pathways associated with progression, survival, and microbial diversity. They showed a higher abundance of *Streptococcus*, correlating with a diminished immune response and reduced life expectancy. Additionally, they revealed an increased abundance of bacteria, including *Streptococcus*, *Prevotella*, and *Veillonella* [90]. Additionally, another study revealed that the lung bacterial burden could predict fibrosis progression, and it showed that microbiota diversity and composition are associated with elevated levels of alveolar inflammatory/profibrotic cytokines, including IL-1Ra IL-1β, CXCL8, MIP-1α, G-CSF, VEGF, and EGF [91].

Alterations in the lung microbiota and their association with histological/tomographic patterns are also essential topics in IPF dysbiosis, but data are controversial in this regard. Lung microbiota composition varies depending on the presence or absence of honeycombing in IPF patients. For example, the enrichment of *Porphyomonas* and *Gemella* and the absence of *Cronobacter* were found in some patients with honeycombing. A subgroup of patients with a higher bacterial load of typical respiratory pathogens, *Streptococcus*, *Veillonella*, and *Prevotella*, had a worse prognosis [92,93].

On the other hand, an imbalance in the gastrointestinal microbiota is linked to the development of lung fibrosis [94]. For example, the relative abundance of *Catenibacterium* and *Lactobacillus* (*L. johnsonii* and *L. gasseri*) is significantly lower in the guts of mice with lung fibrosis induced by bleomycin. In contrast, the relative abundance of *Verrucomicrobia* and *Enterobacteria* significantly increased the apoptosis rate in lung tissue [95]. The results by Shi et al. suggested a possible association between IPF and two microbiota genera: *Blautia* and *Eisenbergiella*. Furthermore, a potential relationship was proposed between IPF and other taxa (*Holdemania*, *Coriobacteriia*, *family XIII*, *Acidaminococcaceae*, *Coriobacteriaceae*, *Hungatella*, *Ruminococcus gnavus*, and *Coriobacteriales*) [96].

Ren et al. explored the causal relationship between gut microbiota and lung function in this context. The results identified four taxa causally linked to the risk of IPF. The order Bifidobacteriales, family *Bifidobacteriaceae*, and *Ruminococcaceae* spp. demonstrated protective effects against IPF, while the genus *Coprococcus 2* was found to promote IPF progression. Several taxa were causally associated with lung function impairment, including the *Lachnospiraceae* family, *Oscillospira* spp., and *Parasutterella* spp. [97].

Thus, alterations in microbiota can impact the progression of pulmonary fibrosis. Developing new strategies to restore normal microbiota could significantly reduce the incidence of age-related diseases. Treatments such as probiotics, microbiota transplantation, and targeted antibiotic use are promising options to restore microbiota balance and enhance health in specific contexts and conditions. A summary is presented in Table 2.

### 2.5. Disabled Macroautophagy

Autophagy encompasses many cellular pathways that are essential for the maintenance of homeostasis through recycling mechanisms. It is involved in the breakdown of specific organelles, protein complexes, protein aggregates, and invading pathogens [98]. Autophagy transports cytoplasmic material of endogenous or exogenous origin to the endolysosomal compartment for enzymatic digestion [99]. Three subtypes can be distinguished: (i) Microautophagy, (ii) chaperone-mediated autophagy, and (iii) macroautophagy. In the latter, cytoplasmic components are sequestered in a double-membraned autophagosome, the distinctive organelle that subsequently fuses with endolysosomal structures to form amphisomes or autolysosomes. This catabolic degradation process, which is tightly controlled by several regulatory units, may be substrate specific or occur non-selectively in response to various situations, e.g., cellular energy deprivation. Macroautophagy is linked to the control of aging and several pathologies, such as IPF [100].

The mTOR kinase is a highly conserved protein that signals the presence of nutrients, promoting cell growth by stimulating ribosomal protein expression and protein translation [101]. mTOR directly prevents autophagy activation, and its activation is involved in the fibrotic phenotype [102]. In human and mouse lung fibroblasts from elders, the expression of autophagy pathway genes is elevated. The disruption of the autophagy flux has been well established in IPF, whereas increased autophagy inhibits the IPF onset. Another indicator of insufficient autophagy in lung fibrosis is the upregulation of p62 and the downregulation of LC3b. These autophagy alterations are mainly involved in the phenotypical alteration of fibroblasts and myofibroblasts [103]. Thus, the induction of autophagy could protect against fibrosis by suppressing inflammation and the EMT [104].

A complex interaction between the lncRNA Taurine Upregulated Gene 1 (*TUG1*) and the cell cycle protein CDC27 has been described in IPF [105]. This lncRNA accelerates the progression of IPF, and its overexpression contributes to fibrosis in various organs, including the liver, heart, and kidney. In TGFβ-treated RLE-6TN cells (AECs from rats), inhibition of *TUG1* prevented EMT, reducing profibrotic markers such as α-SMA, Col-1, and Fibronectin-1. Similarly, in rat pulmonary fibroblasts, the suppression of *TUG1* abolished the TGFβ-induced fibrotic phenotype. The autophagy inhibitor 3-MA reversed this effect. Research indicates that inhibiting CDC27 protects against pulmonary fibrosis by suppressing the PI3K/Akt/mTOR pathway, a process that may be further facilitated by *TUG1* depletion, promoting autophagy. Notably, CDC27 overexpression counteracts the inhibitory effects of *TUG1* suppression on EMT, leading to increased expression of fibrotic markers and activation of the PI3K/Akt/mTOR pathway. The bleomycin-induced rat pulmonary fibrosis model showed a significant decrease in the LC3-II/I ratio and Beclin-1 expression. However, treatment with sh-*TUG1* successfully restored the autophagy deficiency caused by bleomycin. The promotion of autophagy by *TUG1* was attributed to the suppression of CDC27 and the subsequent inactivation of the PI3K/Akt/mTOR pathway [105].

High p62 and reduced LC3BII expression are hallmarks of impaired autophagy in IPF. The mRNA expression of p62 is also increased in the bleomycin model, and TGFβ1 stimulated A549 cells [106]. The administration of FGF21 reduced the mRNA expression of p62 and increased ATG5 and LC3B. Nevertheless, the expression of Beclin-1 was unaffected, and the effects of FGF21 were decreased by adding the autophagy inhibitor 3-MA. Similarly, FGF21 administration reduced the severity of the fibrotic response and increased the apoptosis rate; however, concurrent treatment with 3-MA abated the differences. FGF21 influenced the decrease in fibrotic markers through the phosphorylation of AKT and mTOR, resulting in elevated ATG5 and LC3BII levels, leading to autophagosome formation and finally restoring macroautophagy [106]. Thus, FGF21 enhances autophagy.

**Figure 1:**
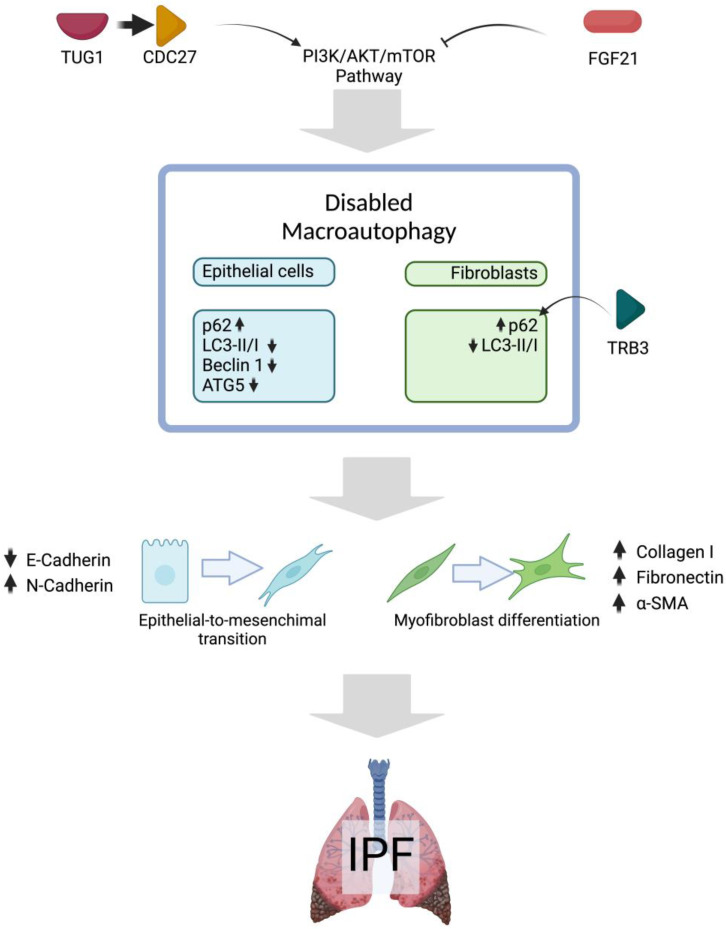
Disabled macroautophagy in pulmonary fibrosis. Evidence from fibrosis animal models and in vitro studies with TGFβ suggests that the PI3K/AKT/mTOR pathway may be activated in IPF, contributing to impaired macroautophagy in both epithelial cells (blue) and fibroblasts (green). Possible consequences of impaired macroautophagy are EMT and fibroblast-to-myofibroblast differentiation, which are considered key processes in the progression of IPF. Upward arrows mean upregulated expression and downward arrow means the opposite. Created in BioRender. Cisneros, J. (2025) https://BioRender.com/u17c477.

In IPF, activated fibroblasts exhibit impaired autophagy, evidenced by the accumulation of the protein p62. This autophagy promotes fibroblast activation and myofibroblast differentiation, driving excessive deposition of ECM [107]. A relationship between p62 and upregulated pseudokinase tribbles homolog 3 (TRB3) suppresses autophagic flux. Using a new strategy for modulating protein–protein interactions (PPIs), modifying the aberrant interaction between p62 and TRB3 was possible. In a model of TGFβ1-activated MRC-5 fibroblasts, the co-localization of TRB3 with p62 was evident, and autophagosomes were increased. However, the accumulation of p62 caused impaired autophagic flux. A supramolecular self-assembly TRB3 peptide was effectively absorbed by MRC-5 cells and could interfere with TRB3/p62 PPIs to restore the autophagic flux, increase the LC3-II: LC3-I ratio, and reduce the levels of the protein p62. The TRB3-binding nanofibers were administered to the lung fibrosis mouse model via intravenous injection, resulting in decreased p62 accumulation and an increased LC3-II/LC3-I ratio. This was accompanied by a subsequent reduction in the fibrotic markers, Collagen 1, Fibronectin, and α-SMA. This approach represents a promising therapeutic strategy for mitigating fibrotic lesions in IPF patients [108]. Figure 1 summarizes disabled macroautophagy in IPF fibroblasts and epithelial cells.

## 3. Classic Hallmarks

### 3.1. Loss of Proteostasis

Protein homeostasis, also known as “proteostasis”, regulates the dynamic equilibrium of functional intracellular proteins. Cell proteome maintenance involves protein initial synthesis and folding, conformational stability, abundance regulation, subcellular localization, and clearance by degradation mechanisms [109].

Proteins participate in several vital processes as catalysts, structural components, and cell cycle regulators. Many pathways are involved in proteostasis regulation, including (1) protein synthesis at ribosomes, (2) protein folding, transport, and processing in the endoplasmic reticulum (ER), and (3) protein degradation through the action of the Ubiquitin–Proteasome System (UPS) and the Autophagy–Lysosome Pathway System (ALPS). Proteostasis pathways rely on biogenesis and degradation machinery, with molecular chaperones coordinating protein folding and conformational maintenance. Notably, loss of proteostasis is associated with the formation of toxic protein aggregates, a phenomenon observed in aging and several medical conditions [110].

Substantial evidence highlights the influence of proteostasis loss on the pathogenesis of IPF and its importance. The following section lists the fundamental mechanisms of proteostasis loss in IPF.

#### 3.1.1. Decline in Protein Quality Control Systems

Nascent polypeptide chains at the ribosome are folded with assistance from ribosome-associated cytoplasmic chaperones. Several families of chaperones are essential in protein assembly and include the Heat Shock Proteins (HSP) HSP100, HSP90, HSP70, HSP60, HSP40, and small HSP families [111]. The HSP families are involved in the abnormal synthesis of massive amounts of collagen and are collagen-specific molecular chaperones whose presence stimulates collagen production [112]. Therefore, they play a vital role in fibrogenesis and fibrosis progression.

Hsp families are being studied as possible therapeutic targets for fibrosis. For example, Hsp47 (encoded by Serpin1) probably plays a role in collagen synthesis [113,114]. In one experimental approach using lung slices stimulated with TGF-β1 to induce fibrogenesis, Serpin1 was silenced with a small interfering RNA (siRNA). The fibronectin secretion decreased, but other aspects of fibrogenesis remained unaffected. Similarly, the knockdown of Hsp47 did not affect collagen secretion [115].

On the other hand, the repression of HSP70 was implicated in the etiology of pulmonary fibrosis through mechanisms mediated by EGFR and Dicer1 miRNAs [116]. Zhou et al. evaluated the effect of increasing the expression of HSP70 using Geranylgeranylacetone (GGA), a nontoxic anti-ulcer drug. GGA treatment in the murine bleomycin-induced pulmonary fibrosis model decreased the expression of profibrotic markers. GGA also suppressed the NF-κB/NOX4/ROS pathway by reversing the effect of TGFβ1, thereby postponing the initiation of the EMT in bronchiolar cells. The above results should be interpreted carefully because the Hsp70 levels also increased in the bleomycin model with no GGA. This underscores the need for a comprehensive understanding of the mechanisms governing this protein’s activity on epithelial cells and fibroblasts in the fibrosing lung [117].

HSP90 maintains proteostasis and cell survival by folding and stabilizing proteins under normal physiological conditions and stress-induced scenarios [118]. HSP90 is responsible for stabilizing and folding the TGFβ receptor (TβR). TβRI and TβRII are clients of Hsp90, thereby implicating it in the TGFβ1 signaling. Thus, inhibition of HSP90 counterbalances the aberrant activation of the TGFβ signaling. Alvespimycin, combined with Oleuropein (OLPN), stimulates proteasome activities. Its efficacy was tested as a potential therapeutic method for disrupting TGFβ signaling in a rat model of lung fibrosis. Accordingly, alvespimycin facilitated the destabilization and exposure of the receptors to ubiquitination-dependent or independent degradation by the OLPN-activated proteasomes. Finally, the combined therapy significantly decreased the tissue expression of p-SMAD2/3, resulting in the mitigation of bleomycin-induced pulmonary fibrosis [119].

In IPF, HSP90 levels are elevated, probably participating in developing a senescent phenotype. In a PCLS from naphthalene-injured INKBRITE (p16^Ink4a^) lungs with senescent characteristics, the inhibition of HSP90 with XL888 eliminated senescent fibroblasts with the p16^INK4a+^, ACTA2^+^, and COL1^+^ markers in a bleomycin lung fibrosis model. In vivo, mice treated with the XL888 inhibitor after the bleomycin injury showed reduced fibrotic remodeling in the lung. In human PCLS, the XL888 treatment decreased the pathologic p16^INK4a+^ ACTA2^+^ and p16^INK4a+^ CTHRC1^+^ fibroblasts. Additionally, XL888 attenuated multiple fibrotic indicators, such as total collagen content [120].

#### 3.1.2. Unfolded Protein Response in the ER

The ER is essential in protein synthesis, folding, and quality control. ER stress results in the accumulation of misfolded proteins and the triggering of the unfolded protein response (UPR). This response restores proteostasis, mediates ER chaperone expression, halts protein translation, and degrades incorrectly folded proteins. Abnormal activation of ER stress and the UPR has been linked to several human diseases, including fibrosis. Growing evidence demonstrates that the ER stress pathways play a vital role in the pathology of IPF, altering type II Alveolar Epithelial Cell (AEC) functions and mechanisms [121].

The UPR is orchestrated by three branches: Protein kinase RNA-like Endoplasmic Reticulum Kinase (PERK), Inositol-Requiring Enzyme 1 (IRE1), and Activating Transcription Factor 6 (ATF6). The three axes coordinate downstream components, initially promoting cellular protection [122]. The ER stress results from abnormalities that overwhelm the average ER performance. Therefore, RNA translation must be reduced to limit the input of nascent proteins in the ER. The PERK transmembrane protein initiates the third branch of the UPR [123].

In distal airways in the fibrotic lung, ATF4 and ATF6 co-localized with the mucin MUC5B [124]. This mucin is usually overexpressed in IPF, often due to genetic variants. The MUC5B gain-of-function promoter variant rs35705950 is a risk factor for developing IPF [124,125]. Using a humanized mouse that overexpressed Muc5b under the control of the human *SFTPC* promoter showed exaggerated fibrosis and mortality in response to bleomycin in single and repeated doses. Furthermore, *SFTPC*-Muc5b AECs had increased expression of Atf4, confirming the relationship between ER stress and lung epithelial cells. Supporting the above, inhibition of the ER stress pathway in the same SFTPC-Muc5b bleomycin model decreased the fibrotic injury. This ER stress inhibition was achieved with ISRIB, an integrated stress response inhibitor. Thus, Muc5b mediates fibrosis by promoting ATF4-mediated ER stress responses [124].

The UPR has been linked to profibrotic macrophage activation, and macrophage accumulation is associated with the pathogenesis of IPF. The Atf6a transcript is expressed in profibrotic macrophages from the lungs and CD14+ circulating monocytes of IPF patients, indicating that ATF6a may prevent the activation of the CHOP-induced apoptotic pathway in macrophages. Nevertheless, the ablation of Atf6a in macrophages in the bleomycin model increases profibrotic macrophages and exacerbates fibrogenesis. No differences in the M1/M2 macrophage functional status were detected in the fibrotic stage. Nevertheless, a noteworthy transient CD38+ CD206+ macrophage population was detected in bleomycin-treated mice, suggesting that the ER plays a role in macrophage differentiation through ATF6a. Moreover, the ATF6a CHOP-induced apoptosis pathway is a viable therapeutic target for profibrotic macrophage elimination [126].

The ER stress results from abnormalities that overwhelm the average ER performance. In lung tissues of IPF patients and bleomycin-induced mouse models, ER stress markers (p-eIF2α, p-IREα, ATF6) and fibrosis markers (α-SMA and Collagen-I) are elevated. Widely used Chinese medicines, the Citrus Alkaline Extracts (CAE), were tested as a therapeutic agent in a bleomycin-induced mouse model of PF and an in vitro tunicamycin (TM)-induced ER stress model in A549 cells. Bleomycin increased ER stress biomarkers such as BiP, ATF3, ATF4, and PERK in lung tissues and A549 cells. In contrast, the CAE treatment downregulated the same biomarker levels. Thus, the ER stress pathway restoration may be an essential target for reversing pulmonary fibrosis [127].

Due to their self-renewal and multilineage differentiation properties, Mesenchymal Stem Cells (MSCs) are widely used in cell therapy research. MSCs secrete the glycoprotein Stanniocalcin 1, reducing oxidative stress, ER stress, and profibrotic factors levels in AECs. Thus, the MSC population in the fibrotic lung could help to reverse the fibrotic phenotype. Conversely, TGFβ1-stimulated A549 cells elicited EMT and ER stress markers, including ATF6, ATF4, XBP-1s, and BiP. Opposingly, the co-culture of A549 cells with MSCs reduced the EMT and significantly decreased the protein expression of XBP-1s, XBP-1u, and BiP. Additionally, the gene expression of E-cadherin was restored, whereas the expression of vimentin was inhibited. Finally, the EMT reduction was achieved through the blockade of the IRE1α/XBP1 pathway [128].

Recently, it has been demonstrated that the Caveolin-1 Scaffolding domain Peptide (CSP) participates in the resolution of fibrotic lesions across various models of experimentally induced lung fibrosis. When the CSP treatment was applied, collagen 1 was degraded by extracellular proteolysis. CSP also reduced the ER stress in TGFβ1-treated IPF lung fibroblasts by restoring the basal levels of IRE1α and BiP. Consequently, the inhibition of IRE1α enhances MMP activity, thus increasing the elimination of ECM. Hence, CSP represents a promising candidate for treating IPF [129].

In the lungs of IPF patients, UPR pathway proteins are elevated, including p-eIF2α, p-IREα, and ATF6. In contrast, a decreased expression of the Peroxisome proliferator-activated receptor-γ Coactivator (PGC-1α) was observed. PGC-1α plays a role in coordinating the gene expression of mitochondrial biogenesis components. In IPF fibroblasts, PGC-1α levels are stably repressed. When PGC-1α was blocked in fibroblasts, MRC-5, p-eIF2α, p-IREα, and ATF6 increased. Opposingly, the overexpression of PGC-1α in fibroblasts treated with TGFβ decreased p-eIF2α, p-IREα, and ATF6, as well as α-SMA and Collagen 1. Supporting these results, the ER stress inhibitor (4PBA) decreased the fibrosis markers α-SMA and Collagen-I. PGC-1α-conditioned knockout mice had significant alveolar damage, structural destruction, and collagen fiber accumulation in their alveolar parenchyma. Treating these mice with 4PBA restituted the expression of ER stress and fibrotic markers. Hence, IPF is highly connected with ER stress and lipid metabolism, generating potential targets for new therapeutic strategies [130].

### 3.2. Stem Cell Exhaustion

The disruption of tissue regeneration mechanisms also characterizes aging. In most organs, a small subpopulation of pluripotent cells can activate regenerative pathways in response to damage. However, several investigations have shown that these cells lose their regenerative capacity as they age. In the airway epithelium, basal cells are the main population of self-renewing stem cells that can give rise to multiple cell types, such as goblet cells, club cells, ciliated cells, tuft cells, PNECs, and ionocytes. They are rich in the expression of Tp63 and Krt5, which are used as cellular markers [131]. On the other hand, within the alveolar niche, AECIIs act as epithelial precursors with a high capacity to self-renew and give rise to AECIs after injury [132]. Evidence has shown that AT2 dysfunction is an early initial event in the development of IPF [133]. Recent reports on stem cell exhaustion in fibrotic lungs are discussed here.

In 2020, two single-cell RNAseq assays identified some of the most abundant cell populations in IPF patients [134,135]. In the work of Adams and collaborators, in IPF patients, there was an increase in an epithelial population called “Aberrant Basaloid Cells”. These cells are p63 and keratin 17 positive, and they also express the *COL1A1* gene and do not express other previously established basal cell markers such as keratin-5 and keratin-15 [134]. Meanwhile, Habermann and collaborators found in IPF patients that the basal cells increased and described a rise in the percentage of keratin-17-positive and keratin-5-negative cells. They also demonstrated that these cells express the transcription factor p63 and the *COL1A1* gene [135].

Some aging hallmarks, like telomere attrition, epigenetic alterations, activation of senescence pathways with the corresponding SASP, and inflammaging, influence the exhaustion of epithelial lung stem cells [136]. Epigenetic changes in stem cells include chromatin reorganization due to histone marks like H3K9me2/3. Some authors consider that inhibiting diacetyl transferases or modification of key metabolites (such as acetyl-CoA) may restore stem cell function [137].

Nowadays, aberrant basaloid cells (AbBa) are widely used to describe a population of epithelial cells expressing epithelial, basal, and mesenchymal markers with a profibrotic phenotype due to the production of ECM. In humans, these cells express the KRT5-/KRT17+ markers. Contrastingly, the KRT5-/KRT17+ markers are expressed in IPF patients. At the same time, the Krt8+ marker increases in the differentiation intermediate stem cells (ADI) in the bleomycin-induced mouse model [138].

Through spatially resolved transcriptomics technology, which enables RNA profiling of intact tissue, it was possible to analyze the dynamic of cellular interactions in the lungs. The data identified 30 factors. Each factor was determined by the expression of covariant genes, with little overlap observed between the genes that contributed to identifying most of the factors. Eleven factors were more prevalent in IPF than in the controls. In IPF, AbBa cells were grouped with cells actively participating in the fibrosing response, such as fibroblasts and myofibroblasts. It was identified that this cluster corresponded to the proximity to the fibroblastic foci. In the specific case of KRT5−/KRT17+ cells, these are usually located at the edges of the fibroblastic foci. In the bleomycin model, 3787 differentially expressed genes were identified. In the fibrotic stage at 21d, a compartment in the borders of fibrotic areas characterized by alveolar epithelium macrophages and *Krt8*+ ADI cells with a regenerative profile was observed.

Interestingly, this group suggests that KRT5−/KRT17+ cells in humans and Krt8+ ADI cells in mice do not overlap. KRT5−/KRT17+ cells were correlated with fibroblasts (*HAS1*-hi) and myofibroblasts, whereas Krt8+ ADI cells showed a weaker correlation with myofibroblasts. However, it is crucial to consider that this observation was based on a single-dose bleomycin model, where fibrosis begins to resolve from day 28. Therefore, it remains possible that the Krt8-positive cells are more related to mechanisms to resolve the fibrotic injury [139].

### 3.3. Altered Intercellular Communication

One mechanism for communication among tissues is the production and reception of microvesicles and exosomes. Exosomal miR-23b-3p and miR-494-3p from lung IPF fibroblasts might induce senescence in epithelial cells due to mitochondrial and DNA damage [140].

Lung tissue, sputum, sera, BAL, and urine from patients with IPF contain extracellular vesicles with profibrotic cargo, with irregular levels of the microRNAs miR-let-7d, miR-29a-5p, miR-181b-3p, and miR-199a-3p [141]. Exosomes also contain long noncoding RNAs (lncRNAs), such as *HOTAIRM1*, produced by aberrantly activated AECs. *HOTAIRM1* binds to miR-30d-3p in lung fibroblasts, preventing inhibition of *HSF1* and resulting in proliferation and differentiation [142]. Another kind of exosome cargo is circular RNAs, which were proposed as an IPF diagnostic (hsa_circ_0044226, hsa_circ_0004099, hsa_circ_0008898) and progression biomarkers (hsa_circ_0044226) [143].

In some cases, IPF patients may develop cancer, and extracellular vesicles from a fibroblast might contain mir-19a, which can increase the proliferation of non-small-cell lung cancer via *ZMYND11* and c-Myc [144]. On the other hand, a recent study of exosomes from IPF patients showed that one of the effects of antifibrotic drugs is the return to normal *FASN* (fatty acid synthase) and *ACSL4* (acyl-CoA-synthetase long-chain family member 4) levels, the mRNAs of which were downregulated due to miR-143-5p and miR-342-5p. So, looking into the de novo fatty acid synthesis pathway in IPF is important [145].

The exosomes can also be antifibrotic: (i) exosomes from alveolar type II cells control macrophage differentiation, favor mitochondrial biogenesis, and enhance oxidative phosphorylation [146], (ii) exosomes from bronchial epithelial cells inhibit WNT5A and WNT10B, modifying their interaction with TGFβ1 [147], (iii) exosomes in the sera of IPF patients are low in miR-30b, which, via Runx1-Spred2, can reduce fibrosis and inflammation in the murine model [148], (iv) miR-142-3p from macrophage exosomes can be taken by AECs and lung fibroblasts, and reduce *TGFβ*, *TβR1*, *COL1A1*, and *COL3A1* [149]. Some therapeutic strategies can be designed by using specific extracellular vesicles. A list of the recently described content of IPF exosomes is in Table 3. Previous findings are available elsewhere [150].

### 3.4. Epigenetic Alterations

Epigenetics studies reversible changes that modulate gene expression with no DNA sequence alterations [151]. These modifications must be faithfully transmitted to daughter cells following cell division. Specific proteins generating and sustaining these epigenetic modifications are altered with age [152,153,154,155,156]. Epigenetic alterations are closely related to other aging hallmarks, leading to their use as biological age predictors, known as epigenetic clocks [157,158].

DNA methyltransferases (DNMTs) are epigenetic modifiers that catalyze the addition of methyl groups to cytosines, a process that can inhibit transcription when it occurs in promoter regions. The DNA methylome also participates in IPF, where increased expression of DNMT3a and DNMT3b has been reported [159]. While aging is generally associated with a global decrease in genomic methylation, localized increases in specific CpG sites linked to aging have also been observed [160,161]. Moreover, a macrophage study has shown that metabolism and epigenetics are closely interconnected [162]. For instance, Col3a1 expression is associated with H3K27me3 (trimethylation of lysine 27 of histone 3), which is related to glutamine levels [163].

In addition, Sirtuin 1 (*SIRT1*), a member of the NAD+-dependent deacetylase family, is reduced in IPF [164]. Sirtuins remove acetyl groups from lysines in histones, thereby altering DNA accessibility and impacting both gene transcription and genome stability [165]. Notably, SIRT1 levels decline with age in murine models [154].

Finally, other epigenetic modifiers, such as SUV39H1 (a histone methyltransferase) and the fat mass and obesity-associated protein (FTO, which demethylates adenosine residues in RNA), also exhibit changes during aging [155,156]. However, whether their expression is altered in IPF remains to be elucidated.

### 3.5. Deregulated Nutrient Sensing

The nutrient-sensing system is a complex network of interlocking signaling pathways centered around insulin and Insulin-like Growth Factor-1 (IGF-1), mTOR, Adenosine 5′-Monophosphate-activated Protein (AMP), AMP-activated Protein Kinase (AMPK), and Sirtuins (SIRT) [5]. In addition to inhibiting tyrosine kinase receptors, one of the current treatments for IPF, nintedanib, also inhibits the mTOR pathway [166].

Clinical trials exploring sirolimus and other agents targeting deregulated nutrient sensing are promising [58]. A recent review discusses mTOR inhibitors as potential therapies [167]. In this context, decreased levels of phosphorylated AMPK have been reported in the murine model of pulmonary fibrosis, where omentin-1 prevents fibroblast activation, and calpain 1 may promote ferroptosis [168,169]. On the other hand, amifostine, a detoxifying drug used clinically for radiation-caused cytotoxicity, inhibits bleomycin-induced pulmonary fibrosis by restoring mitochondrial function and cellular metabolism [170]. Moreover, geneticin reduced collagen deposition because of alterations in the TGFβ pathway and AMPK/SIRT1 [171]. Last year, SIRT1 and SIRT3 were proposed as potential diagnostic biomarkers of IPF [172].

The senolytic compound quercetin is another molecule that could alleviate pulmonary fibrosis by acting on the SIRT1/AMPK signaling pathway. This compound reduced α-SMA, Collagen I, and collagen III levels in the murine model. It also enhanced LC3II/LC3I levels, decreased p62 expression, and increased the number of autophagosomes in lung tissue. Treatment with EX-527, a SIRT1 inhibitor, reversed all effects induced by quercetin [173]. Diet supplementation with Insulin-like growth factor binding protein 2 (IGFBP2) in preliminary experiments with the murine model showed that it can inhibit epithelial senescence and diminish the fibrotic score [174].

### 3.6. Cell Senescence

Cell senescence is the stable arrest of the cell cycle and is crucial during embryonic development [175,176]. It is also present in several adult organism processes, where it may have beneficial (e.g., wound healing) or detrimental effects (e.g., sarcopenia). Not all senescent cells present all the senescence-associated markers. Thus, their identification requires the conjunction of several signs, including the cell cycle arrest, inhibition of apoptosis, increased expression of inhibitors of cyclin-dependent kinases (such as p21 and p16), reduced retinoblastoma (Rb) phosphorylation, β-galactosidase activity, the presence of heterochromatin foci, and accumulation of DNA damage markers such as γH2A.X (histone variant with phosphorylated serine 139 of H2A) [177].

The initial step of senescence is the stable arrest of proliferation, followed by the production of a series of SASP molecules. This secretome can recruit immune cells and/or induce ECM changes, stimulating stem cells to repopulate the tissue [175,176]. Numerous studies have demonstrated that IPF epithelial cells and fibroblasts show morphological features of senescence [178,179,180,181,182]. Several factors known to induce cell senescence are also involved in the pathogenesis of IPF, including mitochondrial dysfunction, telomere shortening, and DNA damage.

Research on cellular senescence in IPF is rapidly expanding. Other sections of this review have already included some noteworthy advances in the field. Blokland and collaborators suggested that IPF fibroblasts might induce epithelial senescence [182]. Subsequently, they stated that ECM derived from IPF fibroblasts does not induce cell senescence in vitro [183]. As mentioned before, it is plausible that the ECM does not induce cell senescence per se but modifies cellular functions such as migration and proliferation [9]. The factor that did induce epithelial senescence was identified as mechanical stretch in the context of mechanical ventilation [13], in addition to mitochondrial alterations such as a decrease in the adenine nucleotide translocase ANT1, which is critical for mitochondrial physiology [184]. Moreover, Su and collaborators showed that peroxiredoxin 3 can restore mitochondria function and block AEC senescence [185]. Additionally, Bone Morphogenetic Protein 4 (BMP4) has been shown to mitigate TGFβ1-induced myofibroblast differentiation and ECM synthesis via Smad1/5/9 and Pink1, thereby reducing impaired mitophagy and cellular senescence in murine lung fibroblasts [186].

Experiments using senolytic therapies to eliminate senescent cells have shown promising results in the murine fibrosis model, decreasing fibrotic markers and increasing pulmonary functions [120,178,179,187]. Additionally, because the senolytic therapy with Dasatinib and Quercetin (D+Q) was approved by the Food and Drugs Administration for other diseases, clinical trials with IPF patients progressed rapidly [188]. The first pilot study reported that intermittent doses of D+Q are safe for IPF patients; however, no statistically significant differences in circulating SASP factors between groups were observed [188]. Optimism for this treatment grew when increased levels of α-Klotho, an antiaging protein, were found in the urine of D+Q-treated IPF patients [189]. Nonetheless, a phase I clinical trial showed that the D+Q group presented almost three times more acute exacerbations than the placebo group, alongside general and gastrointestinal discomfort [190]. Thus, more studies are needed to determine whether this therapy is effective.

### 3.7. Other Hallmarks

**Figure 2:**
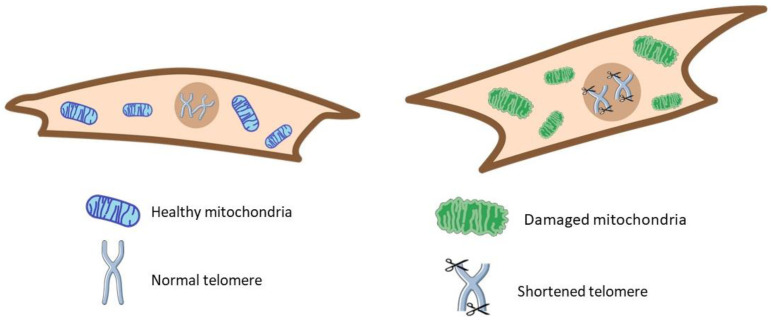
Some classic hallmarks of aging. In IPF (**right**), cells showed shortened telomeres and damaged mitochondria versus the healthy condition (**left**).

One of the first premature aging hallmarks described in IPF was telomere attrition, a risk factor for genomic instability and IPF [191,192,193,194,195]. This hallmark’s complete description and association have been reviewed before [2]. Mitochondrial dysfunction is another critical hallmark described and reviewed elsewhere in IPF [196,197,198,199]. Nevertheless, we included supporting data on mitochondrial alterations in several review sections (Figure 2).

We propose that other cellular hallmarks like nuclear morphology be thoroughly investigated to determine whether they are characteristic of both aging and the pathology of IPF. For instance, age-related nuclear architectural changes encompass alterations in morphology, telomere shortening, heterochromatin loss, and changes in the composition and structure of the nuclear envelope [200]. Therefore, studying nuclear architecture represents a significant research and drug development opportunity.

Potential applications of drugs targeting chromatin structure, such as HDAC inhibitors, should not only rely on gene expression data but also consider changes in the chromatin landscape, nuclear subcompartments, and the overall 3D nuclear architecture. Noncoding RNAs may play a pivotal role as architectural factors, shaping the genome and mediating its functional interactions with proteins specifically localized in subnuclear compartments during the epithelial-to-mesenchymal transition process. Nevertheless, much remains to be studied in IPF [201]. Changes in nuclear architecture and potential implicated ncRNA may play a role in IPF, requiring further evaluation.

**Figure 3:**
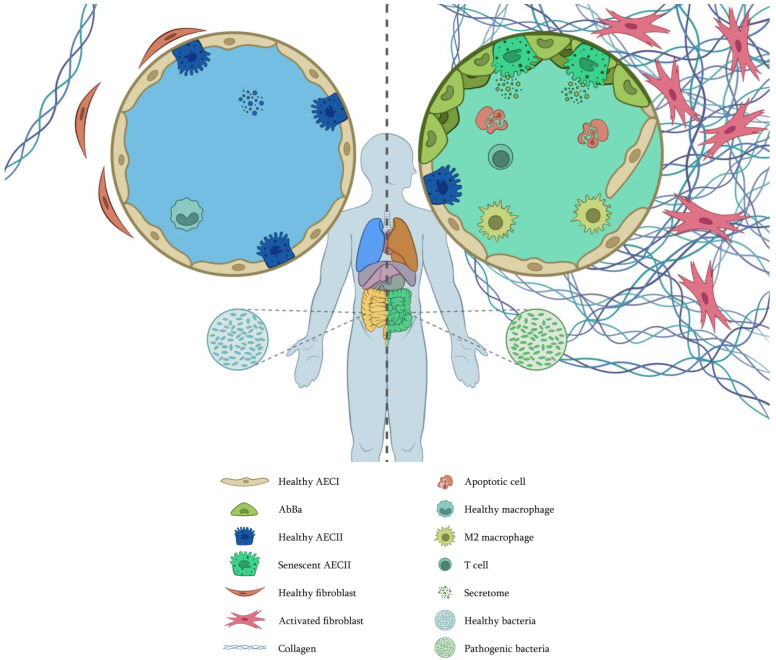
Cellular interplay with altered and premature aging hallmarks in IPF. (**Left**), healthy conditions. (**Right**), IPF. In IPF, AECs have changed; some have died by apoptosis, and many have been replaced by AbBa, compromising the alveolar integrity. Telomere attrition, genomic instability, epigenetic changes, and mitochondrial dysfunction have induced cell senescence. Extracellular vesicles are different, not only in protein content but also in the composition of miRNAs, circRNAs, and lncRNAs. Fibroblasts have been activated, proliferating and secreting vast amounts of extracellular matrix. Fibroblast macroautophagy is also altered, and proteostasis is lost. Macrophages are polarized to an M2 phenotype. T cells are not close to fibrotic foci. In the gut, there are pathogenic bacteria that communicate with the lungs through the vascular system. As there are not enough epithelial stem cells, tissue regeneration is complicated. Image by Víctor Oliveros.

Some of the aging hallmarks in IPF are represented in Figure 3. Recent research suggests that the fibrosing microenvironment influences AbBa cells to remain in a transdifferentiation state by maintaining an altered phenotype and synthesizing ECM components. Therefore, it is imperative to further our knowledge of these cell populations to find new therapeutic strategies that allow them to overcome the altered state, thus leading to tissue regeneration. Moreover, the connection between microbiota and aging also suggests that interventions to restore balance could delay or alleviate age-related diseases. This encompasses not only pulmonary diseases but also metabolic and neurodegenerative disorders, as well as alterations in mitochondrial DNA, considering the impact of inflammation and oxidative stress on mitochondrial damage.

On the other hand, the loss of proteostasis plays a crucial role in developing fibrotic diseases, regulating the production and accumulation of ECM, and participating in other processes like EMT. Some HSP members, like Hsp70, protect against the development of fibrosis, while others contribute to the progression of IPF. Numerous reports have demonstrated the alteration in the macroautophagy flux, and most efforts to find new therapies focus on flux restoration. Key proteins involved in disabled autophagy include p62 and components of autophagosome formation. Thus, increasing knowledge of the regulation and function of these proteins in IPF pathology is essential.

## 4. Translational Research and Summary

Understanding the mechanisms leading to pulmonary fibrosis to identify therapeutic targets and improve drug development processes to achieve effective therapies for patients with IPF is essential. Currently, pirfenidone and nintedanib are the only approved antifibrotic agents for treating IPF. However, these drugs can cause severe secondary effects (e.g., diarrhea, nausea, vomiting, photophobia, and headaches), and we do not entirely know their mechanisms of action. Nonetheless, lung transplantation remains the sole intervention capable of halting the relentless progression of the disease. The above highlights the urgent need for further research and to focus efforts on developing targeted therapies that prolong survival and improve the quality of life of IPF patients. Treatments under evaluation include bexotegrast [16], sirolimus [58], and amifostine [170].

A human recombinant monoclonal antibody targeting the Connective Tissue Growth Factor (CTGF) is being developed as an antifibrotic agent named Pamrevlumab. It is currently in different stages of clinical trials and has been shown to safely and effectively slow the decline of lung function [202]. Treprostinil, a drug approved for pulmonary hypertension, is undergoing Phase III clinical trials (TETON), exhibiting antifibrotic effects through suppressing fibroblast activation and ECM deposition [203]. Additionally, clinical trials such as AETHER and HALT-IPF explore a nonpharmacological approach involving intravenously administered MSCs. This approach has demonstrated safety and promises to reduce disease progression while potentially improving lung function [204]. Additionally, numerous compounds and therapies are being developed and tested as potential treatments for the disease.

**Figure 4:**
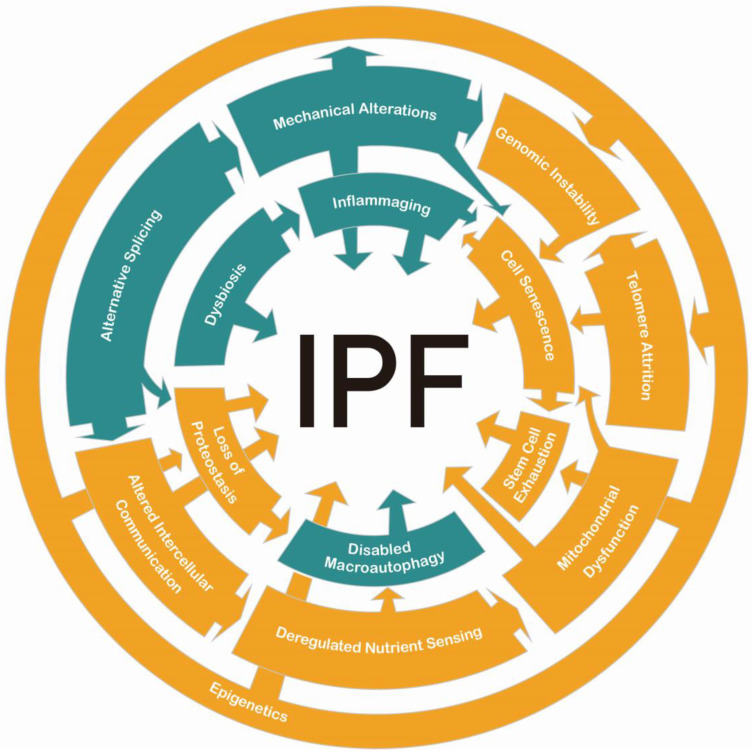
Interactions between aging hallmarks in IPF. In orange are the classic hallmarks. In green are the complementary hallmarks. All of them play pertinent roles in the pathology.

In this review, we describe the most recent advances in aging-associated mechanisms that can be incorporated into those described previously for IPF. Figure 4 shows some of the relationships between the classic and the complementary hallmarks. Continuous research on these topics will clarify if other connections have not been described yet, and new therapeutic targets may be found. However, it is crucial to integrate efforts between basic and clinical research to achieve meaningful progress. Advancements in precision medicine and personalized treatment strategies will be essential for developing more effective, individualized therapies.

Decades of research have delivered advances in the understanding of IPF progression. As discussed, each aging hallmark is prematurely activated and altered in IPF. It is time to look for the causes and consequences of the less-studied hallmarks, the AS, inflammaging, and the gut–lung axis. Broadening our perspective could uncover key events that contribute to IPF development. In addition, it is crucial to continue the study of other interstitial lung diseases that can lead to progressive fibrosis, as some pathways may be shared, whereas others may be specific for IPF. All of these aim to improve the patient’s quality of life.

## Figures and Tables

**Table 2:** Changes in the pulmonary and gut microbiomes associated with IPF disease progression.

*Sample*	*Microbiota Increased in IPF*	*Reference*
*BAL*	*Actinomyces* and *Veillonella*	[86]
*BAL*	*Neisseria*, *Streptococcus*, and *Actinobacterium* sp.	[87]
*BAL*	*Streptococcus*, *Sphingomonas*, *Clostridium*, and *Lactobacillus*	[88]
*Bronchoscopy and BAL*	*Streptococcus* and *Staphylococcus*	[89]
*BAL*	*Streptococcus*, *Prevotella*, and *Veillonella*	[90]
*Human and murine BAL*	*Firmicutes*	[91]
*BAL*	*Prevotella*, *Veillonella*, *Porphyomonas*, *Gemella*, and *Cronobacter*.	[92]
*Bronchial washing and airway tissue*	*Streptococcus*, *Prevotella*, and *Veillonella*	[93]
*Human gut*	*Coprococcus 2*	[94]
*Murine gut*	*Verrucomicrobiales* and *Enterobacteriales*	[95]
*Human gut*	*Blautia* and *Eisenbergiella*	[96]
*Human gut*	*Oscillospira* and *Parasutterella*	[97]

**Table 3:** New profibrotic and antifibrotic cargo (microRNA, circular RNA, and lncRNAs) in IPF extracellular vesicles.

Role	Cargo	Effect	Reference
Profibrotic	miR-143-5p, miR-342-5p	Downregulation of FASN and ACSL-4	[145]
	hsa_circ_0044226	Associated with acute exacerbations	[143]
	hsa_circ_0004099, hsa_circ_0008898	Proposed diagnostic biomarkers	[143]
	HOTAIRM1	Proliferation and differentiation of fibroblasts	[142]
	Low levels of miR-let-7d, miR-29a-5p, and miR-181b-3p and high miR-199a-3p	Increase COL1A1 and α-SMA	[141]
Procancer	mir-19a	Increases proliferation of non-small-cell lung cancer via ZMYND11 and c-Myc	[144]
Antifibrotic	STIM-activating enhancer	Controls macrophage differentiation, enhances oxidative phosphorylation, and favors mitochondrial biogenesis	[146]
	Low miR-30b	Downregulates TNF-α, TβR1, IL-1β, α-SMA, and Collagen 1.	[148]
	miR-142-3p	Downregulates *TGF-β1*, *TβR1*, *COL1A1*, and *COL3A1*	[149]

## Data Availability

No new data were created in this study. Data sharing is not applicable to this article.
